# A probabilistic model of biological ageing of the lungs for analysing the effects of smoking, asthma and COPD

**DOI:** 10.1186/1465-9921-14-60

**Published:** 2013-05-30

**Authors:** Silvia Chiappa, John Winn, Ana Viñuela, Hannah Tipney, Timothy David Spector

**Affiliations:** 1 Microsoft Research Cambridge, , 21 Station Road, Cambridge, CB1 2FB, UK; 2 Department of Twin Research & Genetic Epidemiology, King’s College London, Westminster Bridge Road, London, SE1 7EH, UK; 3 Current address: GlaxoSmithKline Research & Development Ltd, , Gunnels Wood Road, Stevenage, Hertfordshire, SG1 2NY, UK

**Keywords:** Lung function, Biological ageing, Probabilistic model, Generative model, Posterior distributions, Smoking, Asthma, COPD, FEV_1_, FVC

## Abstract

**Background:**

Although a large body of literature is available that describes the effects of smoking, asthma and COPD on lung function, most studies are restricted to a small age range and to one factor. As a consequence, available results are incomplete and often difficult to compare, also due to the ways the effects are expressed. Furthermore, current approaches consider one type of measurement only or several types separately.

**Methods:**

We propose a probabilistic model that expresses the effects as number of years added to chronological age or, in other words, that estimates the biological age of the lungs. Using biological age as a measure of the effects has the advantage of facilitating the understanding of their severity and comparison of results. In our model, chronological age and other factors affecting the health status of the lungs generate biological age, which in turn generates lung function measurements. This structure enables the use of multiple types of measurement to obtain a more precise estimate of the effects and parameter sharing for characterization over large age ranges and of co-occurrence of factors with little data. We treat the parameters that model smoking habits and lung diseases as random variables to obtain uncertainty in the estimated effects.

**Results:**

We use the model to investigate the effects of smoking, asthma and COPD on the TwinsUK Registry. Our results suggest that the combination of smoking with lung disease(s) has higher effect than smoking or lung disease(s) alone, and that in smokers, co-occurrence of asthma and COPD is more detrimental than asthma or COPD alone.

**Conclusions:**

The proposed model or other models based on a similar approach could be of help in improving the understanding of factors affecting lung function by enabling characterizations over large age ranges and of co-occurrence of factors with little data and the use of multiple types of measurement. The software implementing the model can be downloaded at the first author’s webpage.

## Introduction

Smoking, asthma and Chronic Obstructive Pulmonary Disease (COPD) are the primary risk factors for lung function impairment in adults. Their average effects on the lungs are commonly estimated by measuring reduction in spirometric values with respect to a population of healthy individuals [1-7]. Due to the difficulty of collecting large sample size data spanning the entire adulthood, most studies are restricted to a small age range and to one factor. As a consequence, overall ages and combined effects are reported only in a few studies or are still missing and results from multiple studies are often difficult to compare, also due to the ways the effects are expressed. Furthermore, current approaches consider one type of measurement only, or several types separately (mostly Forced Expiratory Volume in 1 second (FEV_1_) or Forced Vital Capacity (FVC)) – a combined analysis of several types of measurement could potentially provide a more precise quantification of the effects.

In this paper we address these issues by taking the viewpoint that reduced pulmonary function corresponds to premature ageing of the lungs: we propose a model that expresses average FEV_1_ and FVC reduction in individuals that smoke and/or have asthma and/or COPD in terms of number of years that are added to the lungs, or, in other words, we propose a model that estimates *biological ageing* of the lungs.

Biological age has been studied mainly at the whole body level (see [8-10] for recent references). At the respiratory system level, it was first introduced in [11] as a potentially more powerful type of information than spirometric values in motivating smokers to quit. Since then, several studies have investigated this hypothesis [12,13], using as biological age of a smoker the chronological age of a non-smoker of same height, gender and average FEV_1_ obtained from predictive equations. This approach was designed to estimate the specific effect of smoking on a single individual rather than the average effect on an entire population, which is the interest of this paper.

We propose a generative probabilistic approach that explicitly represents biological age using an unobserved random variable – an adjustment of chronological age induced by factors that have an impact on the health status of the respiratory system such as smoking habits, lung diseases, environmental and genetic factors, etc. Our *generative* approach enables us to integrate multiple aspects of the problem into a single consistent framework, which allows the use of multiple types of measurement as well as sharing of information and therefore estimation with little data. The *probabilistic* approach enables us to deal with uncertainty and noise in the data. Furthermore, it allows us to treat the parameters that model smoking habits and lung diseases as random variables and therefore to obtain uncertainty in the estimated effects of such factors on the lungs.

We evaluate our model on a subset of the TwinsUK Registry [14]. The dataset contains FEV_1_ and FVC measurements of several individuals along with information about smoking habits, asthma, COPD, and height. By examining the posterior distributions of the parameters that model the combinations of smoking, asthma and COPD, and the posterior distributions representing the biological age associated to each combination, we are be able to make general and age-specific quantitative statements about the effects of these factors.

## Methods

The TwinsUK Registry is a cohort of about 12000 twins aged 16 to 100 years from all over the United Kingdom used to study heritability and genetics of age-related diseases. It includes clinical, physiological, behavioural and lifestyle data collected since 1992 either at visits to the Department of Twin Research at King’s College London or via self-administered questionnaires. For historical reasons, it encompasses predominantly females in the age range 45–65 years.

For the study, we considered female individuals with spirometry data collected between 1992 and 2010 and with recorded height. Males were excluded as their number was too small to enable reliable estimation of model parameters.

The study was approved by the St Thomas’ Hospital Research Ethics Committee, and all twins provided signed informed consent, in accordance with the Helsinki Declaration.

### 

#### FEV_1_-FVC measurements

Spirometry tests (model 2150; Vitalograph; Buckingham, England) were performed during visits (up to five for each individual) to the department. During each test, three FEV_1_-FVC measurements were recorded and the one corresponding to maximum FEV_1_ was selected. The measurements were included in the study if in normal range, identified as between 0.5 and 7.0 litres based on [15,16]. More information can be found in [17].

#### Smoking status

We considered the subset of individuals that responded consistently in different smoking-related questionnaires between 1992 and 2010 (maximum of 13 questionnaires and 52 types of question). For such individuals, only those FEV_1_-FVC measurements for which one of the following two conditions held were included in the study: 

•The individual reported to have never smoked either cigarettes, cigars or pipes in a questionnaire completed in the same (or a subsequent) year in which the measurement was recorded.

•The individual reported to be a smoker in a questionnaire completed in the same year in which the measurement was recorded.

As the same condition was satisfied for all retained measurements from the same individual, an overall-measurement non-smoker or smoker status could be assigned to each individual.

#### Asthma and COPD status

We considered the subset of individuals that responded consistently in different asthma-related questionnaires between 1992 and 2010 (maximum of 8 questionnaires and 4 types of question). Such individuals were classified as non-asthmatic if they reported to have never suffered from asthma and as asthmatic otherwise. Diagnosis by a doctor was not always explicitly required. A similar procedure was used to determine COPD status.

All possible combinations of smoking, asthma and COPD status give rise to 8 groups (see Table 1 where H stands for healthy with respect to smoking, asthma and COPD). Only individuals of known combined status, and therefore group, were included in the study. In order to eliminate potential bias in estimating the effects of smoking, asthma and COPD due to correlation between twins and multiple visits, with the exception of Group H, we disregarded at random one twin for twins belonging to the same group and retained only the most recent FEV_1_-FVC measurement for individuals with multiple visits. Group H, which contains a considerable number of datapoints and should therefore not be heavily affected by this correlation, was excluded as accurate estimation of parameters *b* (see (3)) requires a large amount of data.

**Table 1:** **FEV**_**1**_**-FVC grouping**

	**Non-Smokers**				**Smokers**			
	**No-Asthma**		**Asthma**		**No-Asthma**		**Asthma**	
	**Group H**		**Group A**		**Group S**		**Group SA**	
**No-COPD**	# of Meas.	Age Range	# of Meas.	Age Range	# of Meas.	Age Range	# of Meas.	Age Range
	3742	18.3–82.8	111	19.0–76.5	428	19.0–77.8	17	27.6–74.3
	**Group C**		**Group AC**		**Group SC**		**Group SAC**	
**COPD**	# of Meas.	Age Range	# of Meas.	Age Range	# of Meas.	Age Range	# of Meas.	Age Range
	17	26.2–72.5	18	20.5–66.0	45	21.1–74.3	25	19.4–65.4

Subdivision of FEV_1_-FVC measurements into 8 groups corresponding to all possible combinations of smoking, asthma, and COPD status. For each group, we indicate the number of available measurements and the age range of the associated individuals.

**Figure 1:**
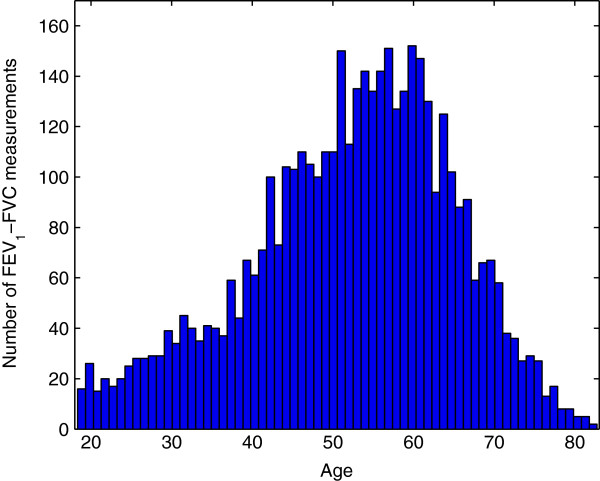
**FEV**_**1**_**-FVC measurements.** Histogram of the number of FEV_1_-FVC measurements available at each age.

These filtering steps are summarized in Table 2. The final dataset encompassed 4403 FEV_1_-FVC measurements taken from individuals in the age range 18.3–82.8 years (the age of an individual, calculated from birth date and date of measurement, is expressed in decimals of year by considering 365.25 days per year). The total number of measurements and the age range of each group are indicated in Table 2. The histogram representing the number of FEV_1_-FVC measurements available at different ages is given in Figure 1. The number of measurements available for Group H at age ranges 18–44, 45–64 and 65–83 is respectively 871, 2221 and 650.

**Table 2:** Data filtering

									
**Total # of FEV**_**1**_**-FVC measurements**	11943								
**Less males**	11280								
**Less individuals of unknown height**	10885								
		**Groups**							
		**H**	**A**	**C**	**AC**	**S**	**SA**	**SC**	**SAC**
**Less individuals of unknown, inconsistent, etc., status**	4735	3742	177	19	25	672	18	54	28
**Less multiple visits**	4489	3742	126	17	19	491	17	50	27
**Less half twins (for twins in the same group)**	4403	3742	111	17	18	428	17	45	25

Description of the filtering steps applied to the available FEV_1_-FVC measurements giving rise to the 4403 measurements used in the study.

Our classification does not take into account the degree of severity of asthma, COPD and smoking. Therefore, the estimated effects have to be interpreted as corresponding to the most likely degree. We are also limited by our definition of asthma and COPD, which potentially includes individuals with a self-reported diagnosis. Finally, whilst the definition of non-smoker and smoker is based on the year in which the FEV_1_-FVC measurement was taken, this is not the case for asthma and COPD, as we do not have precise timing information about these diseases. We nevertheless expect little error due to this as each individual answered the questionnaires multiple times.

### Definition of biological age

Before describing the proposed model in details, we define biological age and highlight key points that guided us in the construction of the model.

**Figure 2:**
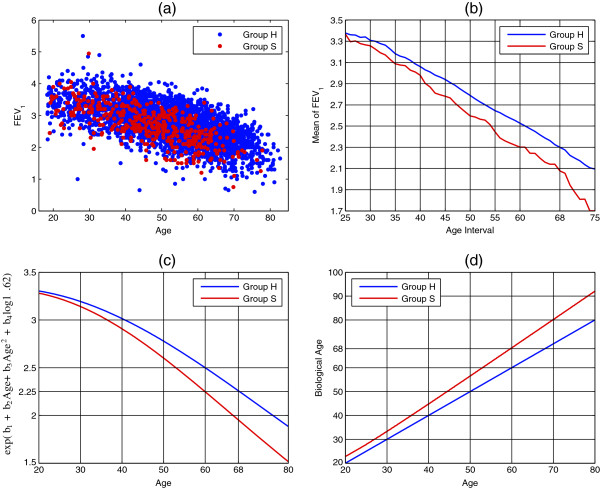
**FEV**_**1**_** of healthy individuals and smokers.** (**a**): FEV_1_ measurements (in litres) of healthy individuals (Group H, blue) and smokers (Group S, red). (**b**): Mean change of FEV_1_ for healthy individuals and smokers over ages. To enforce smoothness, each mean value is calculated over an age interval of 11 years (the X-axis labels indicate the ages at the middle of the intervals). (**c**): Estimated average decline of FEV_1_ for healthy individuals and smokers of average height (1.62 metres) using the model defined by (1). (**d**): Biological ageing of smokers relative to healthy individuals inferred from (c) using as definition of biological age the chronological age of healthy individuals with equal lung function mean.

Figure 2 illustrates the concept of biological ageing for smokers (Group S) relative to the reference population of healthy individuals (Group H) based on FEV_1_. As we can see from the measurements (Figure 2(a)), smokers have on average lower FEV_1_ than healthy individuals. This becomes clearer when looking at the measurement means (Figure 2(b)), which are averages computed over an 11-year sliding window to enforce smoothness over ages. For example, smokers’ mean at age 60 (computed from age interval 55–65) is equal to that of healthy individuals at age 68. It is therefore reasonable to define smokers’ biological age at chronological age 60 as approximately 68 years. That is, biological age is defined to be the chronological age of the healthy population corresponding to the same lung function mean. This is the population level analogue of the individual level definition introduced in [11-13].

A straightforward approach to estimating biological ageing would be to compute differences in average FEV_1_ decline between healthy individuals and smokers by fitting two separate lung function models (such a separate approach was used for example in [1,18]), and subsequently deduce biological ageing from these differences. We can use, for example, the model in [15] first proposed in [19], which is considered an accurate predictor of lung function in adults. In this model, the relationship between the log of the *n*th lung function measurement, *l*^*n*^, chronological age, *a*^*n*^, and height, *h*^*n*^, is given by the following equation:

(1) $$\begin{array}{ll} l^{n}=b_{1}+b_{2}a^{n}+b_{3}{\left(a^{n}\right)}^{2}+b_{4}\log h^{n}\;, & \; \end{array}$$

where *b*={*b*_1_,*b*_2_,*b*_3_,*b*_4_} is a set of unknown model parameters (modelling the log of the measurement, rather than the measurement, makes the model linear in *b* and therefore simplifies its estimation). By computing two separate sets *b*, one for healthy individuals and one for smokers, we can obtain the average FEV_1_ decline for the two populations, as shown in Figure 2(c) for individuals of average height (1.62 metres). From such estimates we can deduce smokers’ biological ageing, as shown in Figure 2(d).

This simple approach has several limitations. It cannot produce reliable estimates of *b* for the groups of small size (all groups other than Groups H and S). A single model of all groups in which some parameters are shared among them would alleviate this problem. Linear regression models that include factors such as smoking and lung disease as covariates, e.g. [20], have this property but are limited to additive combinations of effects.

**Figure 3:**
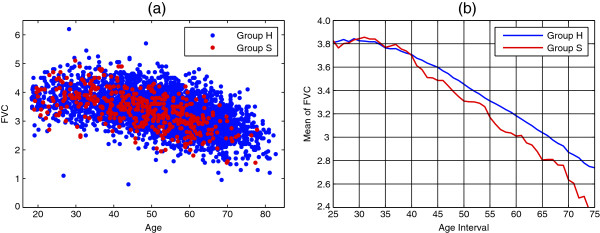
**FVC of healthy individuals and smokers.** (**a**): FVC measurements (in litres) of healthy individuals (Group H, blue) and smokers (Group S, red). (**b**): Mean change of FVC for healthy individuals and smokers over ages. To enforce smoothness, each mean value is calculated over an age interval of 11 years (the X-axis labels indicate the ages at the middle of the intervals).

Furthermore, it is not clear how to consider multiple types of measurement, such as FEV_1_ and FVC, to obtain a more precise estimate of biological age. If two separate models for FEV_1_ and FVC are fitted, the inferred biological ages need to be combined into a single estimate. Simply taking the average (as investigated in [11]) is not optimal as for example, for young ages for which differences between healthy individuals and smokers are absent in FVC (see Figure 3), only FEV_1_ should be considered. An approach that estimates biological age from simultaneous modelling of FEV_1_ and FVC would overcome this difficulty.

Finally, a probabilistic approach would better deal with noise in the data and would allow to obtain uncertainty in the estimated biological ages, which is particularly important when little amount of data is available.

### A probabilistic model of biological age

Our approach to taking into account the observations above is to define a probabilistic model with an explicit *unobserved* random variable representing biological age. This variable is an adjustment of chronological age due to smoking habits, lung diseases, environmental and genetic factors, etc., namely all factors that have an impact on the health status of the respiratory system. Biological age combined with other factors that do not affect the health status of the respiratory system but heavily influence lung function measurements, namely height and measurement noise, generate FEV_1_ and FVC.

More specifically, our probabilistic model is defined by the following equations:

(2) $$\begin{array}{c} {ã}^{n}=u_{c^{n}}a^{n}+v_{c^{n}}+\epsilon^{n},\epsilon^{n}∼N(0,\sigma_{ã}^{2}),\;\\ \;u_{c^{n}}∼N(1,10000),v_{c^{n}}∼N(0,10000)\;,\; \end{array}$$

(3) $$\begin{array}{cc} l^{n}=b_{1}+b_{2}{ã}^{n}+b_{3}{\left({ã}^{n}\right)}^{2}+b_{4}\log h^{n}+\eta^{n},\; & \;\\ \;\eta^{n}∼N(0,\Sigma_{l})\;.\; \end{array}$$

In these equations, *l*^*n*^ is a two-dimensional column vector containing the log of the *n*th FEV_1_-FVC measurement (*n* indexes the measurement rather than the individual, as in Group H each individual can have more than one measurement), *a*^*n*^ is the chronological age of the corresponding individual, *h*^*n*^ is the height, *ã*^*n*^ is the biological age, *c*^*n*^ is a discrete variable representing the group to which measurement *n* belongs (*c*^*n*^∈{1,…,8} corresponding to {Group H, Group A, Group C, Group AC, Group S, Group SA, Group SC, Group SAC}), and $\sigma_{ã}^{2}$, *b*_*i*_ (*i*=1,…,4) and *Σ*_*l*_ are unknown deterministic parameters.

Biological age *ã*^*n*^ is generated as a group-dependent linear transformation of chronological age *a*^*n*^, $u_{c^{n}}a^{n}+v_{c^{n}}$, with the addition of a Gaussian term *ϵ*^*n*^. The term *ϵ*^*n*^ represents the modification to chronological age that is specific to the *n*th measurement and not captured at the group level, and therefore also includes all unmeasured factors such as environmental and genetic factors.

Log-measurement *l*^*n*^ is obtained as a nonlinear transformation of biological age *ã*^*n*^ and height *h*^*n*^ (of the same form as (1)), to which a Gaussian noise term *η*^*n*^ is added. The term *η*^*n*^ is drawn from a two-dimensional Gaussian with non-diagonal covariance matrix *Σ*_*l*_, which accounts for the high correlation between FEV_1_ and FVC. The parameters *b*_*i*_ (*i*=1,…,4) are two-dimensional column vectors that model age-related decline of FEV_1_ and FVC. They are estimated from healthy individuals only to ensure that they describe lung function decline in the absence of smoking, asthma and COPD. These parameters are common to all groups, which is crucial in enabling the inclusion of groups with a small number of available datapoints.

**Figure 4:**
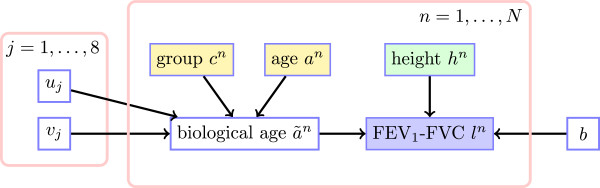
**Probabilistic model of biological age.** Generative process induced by our model. The two plate sections indicate that the enclosed structures are repeated for all 8 groups and *N* measurements. Combined smoking, asthma and COPD status *c*^*n*^ (through parameters $u_{c^{n}},v_{c^{n}}$ and the addition of a noise term *ϵ*^*n*^ representing influence of smoking habits, lung disease, and unmeasured factors such as environmental and genetic factors that are specific to the *n*th measurement) transforms chronological age *a*^*n*^ into biological age *ã*^*n*^. Biological age *ã*^*n*^ and height *h*^*n*^ generate (through parameters *b* and the addition of a noise term *η*^*n*^ representing measurement noise) lung function measurement *l*^*n*^.

The generative process induced by the model is depicted in Figure 4, where empty nodes indicate unknown quantities, whilst filled nodes indicate known quantities.

The linear transformation of chronological age contains both a slope $u_{c^{n}}$ and an intercept $v_{c^{n}}$. The slope $u_{c^{n}}$ determines the rate at which biological age changes with chronological age. Only positive values of $u_{c^{n}}$ are to be expected as they indicate that biological age *increases* with chronological age: $u_{c^{n}}=1$ indicates an increase rate of one year per year, whilst $u_{c^{n}}>1$ (<1) indicates an increase rate higher (lower) than one year per year. For example, Figure 2(d) implies *u*_5_>1. The intercept $v_{c^{n}}$ determines the value of biological age at birth.

Parameters *b*_*i*_ (*i*=1,…,4), $\sigma_{ã}^{2}$ and *Σ*_*l*_ are treated as deterministic quantities and their values are learned as detailed in the Appendix. Parameters *u*_*j*_ and *v*_*j*_ (*j*=1,…,8) are treated as independent Gaussian random variables. This enables us to obtain uncertainty in the estimated effects of smoking, asthma and COPD. The large variance makes the prior uninformative, which ensures that the posterior variance, and therefore uncertainty in the estimated effects, fully depends on the data.

In a probabilistic formulation, we can write the model as

$$\begin{array}{lll} & \;p(u_{c^{n}},v_{c^{n}}|\mu ,\Sigma )=N(\mu ={\left(1,0\right)}^{\text{T}},\Sigma =10000I)\;,\; & \;\\ & \;p({ã}^{n}|a^{n},c^{n},u_{c^{n}},v_{c^{n}},\sigma_{ã}^{2})=N(u_{c^{n}}a^{n}+v_{c^{n}},\sigma_{ã}^{2})\;,\; & \;\\ & \;p(l^{n}|{ã}^{n},h^{n},b,\Sigma_{l})=N(b_{1}+b_{2}{ã}^{n}+b_{3}{\left({ã}^{n}\right)}^{2}+b_{4}\log h^{n},\Sigma_{l})\;,\; & \; \end{array}$$

where the symbol ^T^ indicates the transpose operator and *I* is the identity matrix. To simplify the notation, in the rest of the paper we omit conditioning on all quantities that are not treated as random, namely *μ*, *Σ*, *a*^*n*^, *c*^*n*^, $\sigma_{ã}^{2}$, *h*^*n*^, *b*, *Σ*_*l*_, and therefore denote the three basic Gaussian density functions defining the model as $p(u_{c^{n}},v_{c^{n}})$, $p({ã}^{n}|u_{c^{n}},v_{c^{n}})$ and *p*(*l*^*n*^|*ã*^*n*^).

#### Inference

In order to make deductions about the effects of smoking, asthma and COPD, we need to infer the posterior distributions of the group parameters given all *N* measurements, *p*(*u*_*j*_,*v*_*j*_|*l*^1^,…,*l*^*N*^) (*j*=1,…,8), and the posterior distributions describing the biological age of each group at chronological age *a*, *p*(*u*_*j*_*a*+*v*_*j*_|*l*^1^,…,*l*^*N*^). An analysis of *p*(*u*_*j*_,*v*_*j*_|*l*^1^,…,*l*^*N*^) enables us to make *general (summarized over all ages)* statements about the groups: lack of or small overlap of some of these distributions indicates fundamentally different biological ageing of the corresponding groups. An analysis of *p*(*u*_*j*_*a*+*v*_*j*_|*l*^1^,…,*l*^*N*^) enables us to make statements which are *specific to age a*.

As explained above, we treat *u*_*j*_ and *v*_*j*_ as a priori independent random variables with Gaussian distributions. The joint posterior distribution factorizes as

$$\begin{array}{lll} \;\;\;\;p(u_{1},\ldots ,u_{8},v_{1},\ldots ,v_{8}|l^{1},\ldots ,l^{N}) & =\overset{8}{\underset{j=1}{\prod }}p(u_{j},v_{j}|\{l^{n}|c^{n}=j\})\;,\; & \; \end{array}$$

where {*l*^*n*^|*c*^*n*^=*j*} denotes the subset of measurements belonging to group *j*. The factors *p*(*u*_*j*_,*v*_*j*_|{*l*^*n*^|*c*^*n*^=*j*}) have unknown analytical form, as the transformation from the biological age to the measurements (3) is nonlinear. We estimated them numerically and found that they are all indistinguishable from Gaussian density functions. As a consequence, we also found that *p*(*u*_*j*_*a*+*v*_*j*_|{*l*^*n*^|*c*^*n*^=*j*}) are Gaussian. A detailed explanation of how to estimate these posterior distributions is given in the Appendix

## Results

In the next two sections we analyse the posterior distributions *p*(*u*_*j*_,*v*_*j*_|{*l*^*n*^|*c*^*n*^=*j*}) and *p*(*u*_*j*_*a*+*v*_*j*_|{*l*^*n*^|*c*^*n*^=*j*}) obtained when fitting the proposed model to our dataset.

### Analysis of posterior distributions *p*(*u*_*j*_,*v*_*j*_|{*l*^*n*^|*c*^*n*^=*j*})

**Figure 5:**
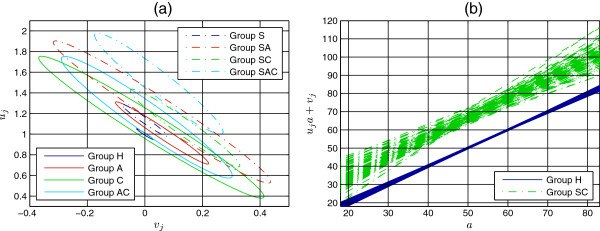
**Posterior distributions*p*(*u***_***j***_**,*v***_***j***_**|{*l***^***n***^**|*c***^***n***^**=*j*}).** (**a**): Contour plots of the posterior distributions *p*(*u*_*j*_,*v*_*j*_|{*l*^*n*^|*c*^*n*^=*j*}). For each group, we show an ellipse centred at the mean and enclosing 95% of the distribution. (**b**): Linear transformation of chronological age *a*, *u*_*j*_*a*+*v*_*j*_, for 100 pairs (*u*_*j*_,*v*_*j*_) sampled from *p*(*u*_*j*_,*v*_*j*_|{*l*^*n*^|*c*^*n*^=*j*}) for Groups H (continuous blue) and SC (dashed-green), showing that uncertainty is higher at young and old ages and lower at middle ages.

Figure 5(a) shows the contour plots of *p*(*u*_*j*_,*v*_*j*_|{*l*^*n*^|*c*^*n*^=*j*}): each ellipse is centred at the mean and encloses 95% of the distribution.

We can notice that the posterior distributions have different spread, depending on the combined effect of number and dispersion of measurements. For Group H (continuous-blue ellipse), the high number of available measurements makes the distribution highly peaked around *u*_1_=1, *v*_1_=0, despite the high dispersion at each age (see Figure 2(a) and Figure 3(a)). This highlights an important point about how to interpret the posterior distributions: they provide us with a measure of uncertainty on the estimated *average* biological ageing. Thus, even if dispersion at each age is high, the model can still be certain about the average biological age.

The major axes of the ellipses all have very similar directions, expressing the fact that increasing the slope *u*_*j*_ requires decreasing the intercept *v*_*j*_ and vice-versa. This means that samples from the posterior distributions give linear transformations of chronological ages intersecting at middle ages, as shown in Figure 5(b) for Groups H and SC. In other words, uncertainty about biological age is higher at young and old ages than at middle ages, which is what we would expect from the distribution of measurements shown in Figure 1.

With the exception of Group C (continuous-green ellipse) for which there is small overlap, unhealthy groups do not overlap with Group H indicating that biological ageing differs from chronological ageing.

If we consider Group A (continuous-red ellipse) versus Group SA (dashed-red ellipse), Group C versus Group SC (dashed-green ellipse), and Group AC (continuous-cyan ellipse) versus Group SAC (dashed-cyan ellipse), we can see that the ellipses do not overlap (considerably) and that the centre of the smoking ellipse is closer to the upper-right corner than the centre of the non-smoking ellipse, which means that smoking in addition to having lung disease(s) induces significant increase in ageing with respect to having lung disease(s) alone. The fact that Group S (dashed-blue ellipse) does not overlap with Groups SA, SC and SAC and is closer to the lower-left corner signifies that this increase in ageing is not due to smoking alone but is a truly *combined* effect. We can therefore conclude that the combination of smoking with lung disease(s) has more severe effect on ageing than lung disease(s) alone. Lack of overlap despite the very small number of available measurements, which causes considerable spread of some of these distributions, makes us confident about this conclusion.

Comparison of Groups A and C with Group AC and comparison of Groups SA and SC with Group SAC reveal the effect of co-occurrence of asthma and COPD versus either disease. Unlike the non-smoking case for which the large overlap does not enable us to draw conclusions, in the smoking case the posterior distributions indicate substantial increase in ageing in the co-occurrence of the diseases.

### Analysis of posterior distributions *p*(*u*_*j*_*a*+*v*_*j*_|{*l*^*n*^|*c*^*n*^=*j*})

**Figure 6:**
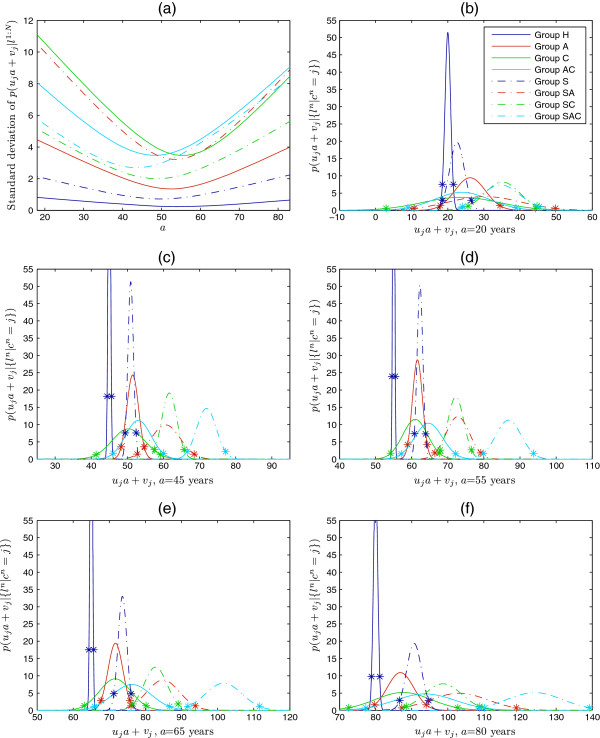
**Posterior distributions*p*(*u***_***j***_***a*+*v***_***j***_**|{*l***^***n***^**|*c***^***n***^**=*j*}).** (**a**): Standard deviations of the posterior distributions *p*(*u*_*j*_*a*+*v*_*j*_|{*l*^*n*^|*c*^*n*^=*j*}). (**b**-**f**): Posterior distributions *p*(*u*_*j*_*a*+*v*_*j*_|{*l*^*n*^|*c*^*n*^=*j*}) for ages *a*=20, 45, 55, 65 and 80 years. The length between two starts equals 2 ×1.96 times the standard deviation. The legend in (b) is valid for all plots.

**Figure 7:**
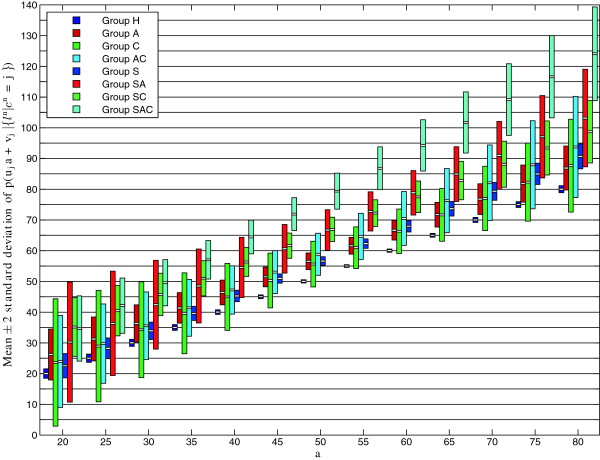
**Posterior distributions*p*(*u***_***j***_***a*+*v***_***j***_**|{*l***^***n***^**|*c***^***n***^**=*j*}) over all ages.** Posterior distributions *p*(*u*_*j*_*a*+*v*_*j*_|{*l*^*n*^|*c*^*n*^=*j*}) for age *a* in the range 20–80 years at 5-year step-size. Each rectangle is centred at the mean and its length equals 2 ×1.96 times the standard deviation.

Figure 6(a) shows the standard deviations of *p*(*u*_*j*_*a*+*v*_*j*_|{*l*^*n*^|*c*^*n*^=*j*}). As discussed above, the standard deviations, and therefore uncertainties about the estimated effects, are lower at middle ages for which more measurements are available. Figure 6(b-f) show the posterior distributions *p*(*u*_*j*_*a*+*v*_*j*_|{*l*^*n*^|*c*^*n*^=*j*}) at ages 20, 45, 55, 65 and 80 years: the length between two starts equals 2 ×1.96 times the standard deviation. Figure 7 illustrates the behaviour of the posterior distributions every 5 years: each rectangle is centred at the mean and its length equals 2 ×1.96 times the standard deviation.

From these figures we can see that, at the extreme ages of 20 and 80 years for which the standard deviations are higher, some of the general conclusions made in the previous section are no longer valid. More specifically, at age 20 there is considerable overlap between Groups A and SA, between Groups C and SC, and between Groups AC and SAC. Therefore, it is not possible to deduce from the posterior distributions that the combination of smoking with lung disease(s) has more severe effect on ageing than smoking or lung disease(s) alone at this early age. Similarly, we cannot make conclusions about co-occurrence of asthma and COPD versus either disease. At age 80, Groups AC and SAC are significantly different, as are Groups S and SAC, so that we can conclude that the combination of smoking with asthma-COPD (with asthma-COPD we indicate co-occurrence of asthma and COPD) has more severe effect on ageing. However, this is not the case for asthma and COPD alone. Furthermore, we cannot conclude that the combined effect of asthma and COPD is higher than the single effects. By looking at the other ages, we can see that the full set of statements made in the previous section is valid for the age range 50–60.

Notice that the difference between Groups H and S is already significant at age 30. This shows that at young ages the model is considering FEV_1_ measurements only to determine smokers’ biological age, as desired (see discussion of Figure 3 above).

This age-specific analysis has enabled us to determine at which ages the general statements about differences in groups made in the previous section are valid. However, it also reveals an important difference between younger and older ages, namely that, with the exception of Groups A and C, means distances of unhealthy groups from Group H are substantially higher at older ages. Thus the effects of most combinations of factors seem to increase with age.

In Table 3 we give the estimated number of years that are added to chronological age (means ±1× standard deviations) for the age range 45–64. From the table we can make a final interesting observation: at age 50 the effect of combined smoking with asthma-COPD seems more severe than additive. Indeed, when considering 1.96 times the standard deviation, the sum of the maximum numbers of years added to chronological age in Groups S and AC is 23.8, whist the minimum number of years added in Group SAC is 23.6.

**Table 3:** Estimated number of years added to chronological age

	**Age 45**	**Age 46**	**Age 47**	**Age 48**	**Age 49**	**Age 50**	**Age 51**	**Age 52**	**Age 53**	**Age 54**
**Group A**	6.6 ±1.7	6.6 ±1.7	6.6 ±1.6	6.6 ±1.5	6.6 ±1.5	6.6 ±1.4	6.7 ±1.4	6.7 ±1.4	6.7 ±1.4	6.7 ±1.4
**Group C**	5.3 ±4.8	5.4 ±4.6	5.5 ±4.4	5.5 ±4.3	5.6 ±4.1	5.7 ±4.0	5.7 ±3.9	5.8 ±3.8	5.9 ±3.7	5.9 ±3.6
**Group AC**	8.0 ±3.7	8.2 ±3.6	8.4 ±3.6	8.5 ±3.6	8.7 ±3.5	8.8 ±3.5	9.0 ±3.6	9.2 ±3.6	9.3 ±3.7	9.5 ±3.7
**Group S**	5.9 ±0.8	6.1 ±0.8	6.2 ±0.8	6.3 ±0.7	6.5 ±0.7	6.6 ±0.7	6.7 ±0.7	6.9 ±0.7	7.0 ±0.7	7.1 ±0.8
**Group SA**	15.7 ±4.2	15.9 ±4.1	16.2 ±3.9	16.4 ±3.8	16.6 ±3.6	16.8 ±3.5	17.0 ±3.4	17.2 ±3.4	17.5 ±3.3	17.7 ±3.3
**Group SC**	16.9 ±2.2	16.9 ±2.1	17.0 ±2.1	17.1 ±2.1	17.1 ±2.0	17.2 ±2.0	17.2 ±2.0	17.3 ±2.1	17.4 ±2.1	17.4 ±2.1
**Group SAC**	27.0 ±2.8	27.5 ±2.8	28.0 ±2.8	28.5 ±2.8	29.0 ±2.9	29.5 ±3.0	29.9 ±3.1	30.4 ±3.1	30.9 ±3.3	31.4 ±3.4
	**Age 55**	**Age 56**	**Age 57**	**Age 58**	**Age 59**	**Age 60**	**Age 61**	**Age 62**	**Age 63**	**Age 64**
**Group A**	6.7 ±1.4	6.7 ±1.4	6.7 ±1.4	6.7 ±1.5	6.7 ±1.5	6.8 ±1.6	6.8 ±1.7	6.8 ±1.7	6.8 ±1.8	6.8 ±1.9
**Group C**	6.0 ±3.6	6.1 ±3.5	6.1 ±3.5	6.2 ±3.6	6.3 ±3.6	6.3 ±3.7	6.4 ±3.7	6.5 ±3.9	6.5 ±4.0	6.6 ±4.1
**Group AC**	9.7 ±3.8	9.8 ±3.9	10.0 ±4.0	10.2 ±4.1	10.3 ±4.3	10.5 ±4.4	10.6 ±4.5	10.8 ±4.7	11.0 ±4.9	11.1 ±5.0
**Group S**	7.3 ±0.8	7.4 ±0.8	7.6 ±0.8	7.7 ±0.9	7.8 ±0.9	8.0 ±0.9	8.1 ±1.0	8.2 ±1.0	8.4 ±1.1	8.5 ±1.1
**Group SA**	17.9 ±3.3	18.1 ±3.3	18.3 ±3.3	18.5 ±3.4	18.7 ±3.5	19.0 ±3.6	19.2 ±3.7	19.4 ±3.9	19.6 ±4.0	19.8 ±4.2
**Group SC**	17.5 ±2.2	17.5 ±2.3	17.6 ±2.3	17.7 ±2.4	17.7 ±2.5	17.8 ±2.6	17.8 ±2.7	17.9 ±2.8	18.0 ±2.9	18.0 ±3.0
**Group SAC**	31.9 ±3.5	32.4 ±3.6	32.9 ±3.7	33.4 ±3.9	33.9 ±4.0	34.4 ±4.2	34.8 ±4.3	35.3 ±4.5	35.8 ±4.6	36.3 ±4.8

Number of years added to chronological age (means ±1× standard deviations) by the combinations of smoking, asthma and COPD as estimated by the proposed model.

## Discussion

To date, biological age of the lungs has been used at the individual level to investigate its effectiveness in motivating smokers to quit. In this paper, we have used biological age of the lungs at the population level to analyse the average effects of smoking, asthma and COPD on the health status of the respiratory system. As for the individual level case, knowing how much older, on average, the lungs of individuals that smoke and/or have lung disease(s) look relative to the healthy population enables a more immediate understanding of the impact of these factors on the health status of the lungs. However, with this work we have shown that modelling lung function through biological age has additional benefits.

Such a modelling enables to properly combine multiple types of measurement to obtain a more precise estimate of the health status of the respiratory system. We have seen that our approach correctly deals with the case in which lung function differences are not evident in one type of measurement.

Such a modelling also enables parameter sharing for characterization over large age ranges and of co-occurrences of factors with little data. We obtained results that are in agreement with the literature (see the next section) using a small amount of data. Furthermore, we could compare cases that have not been previously analysed, as non-smokers with asthma and COPD versus smokers with asthma and COPD.

By treating the parameters that model smoking and lung diseases as random variables, we could obtain uncertainty in the estimated effects of such factors on the lungs.

Finally, such a modelling enables more immediate interpretation and comparison of results within and among different studies than approaches expressing effects in spirometric values. Whilst we did not show that in this paper, the following examples can clarify this point. Suppose that Studies A and B find that FEV_1_ mean value at age 60 in the healthy population is respectively 2.75 and 2.5 litres, and that both studies find that FEV_1_ mean value at age 60 in the smoking population is 2.25 litres. One has to consider the mean values of the healthy populations to understand that Study A estimates that smoking has a stronger effect than Study B. On the other hand, this would be immediately evident if biological age was used, since the estimated number of years added to chronological age in smokers in Study A would be higher than in Study B. As another example, consider investigating whether the effect of smoking on pulmonary function in females and males is different (published results on this subject are controversial [21-27]). Whilst our analysis was restricted to females, males can be easily included in the model e.g. by having separate sets of parameters *b*, *u* and *v* so that only noise covariances are shared between genders. Similarly to the previous example, if spirometric values are compared as in current studies, the values of healthy males and females need to be considered to understand whether the impact of smoking is gender specific, whilst this is not the case if biological age is used, as biological age is a measure that is relative to the healthy population.

One limitation of the proposed model is that it does not account for longitudinal and twin structure, so that we had to exclude many datapoints from the analysis. We are currently investigating an extension that incorporates both types of structure by adding Gaussian terms which are shared across ages and twins.

The choice of modelling biological age as a linear transformation of chronological age, as defined in (2), was motivated by simplicity and supported by Figure 2(d). This figure indicates that smokers’ biological age is well described as a linear transformation and makes it reasonable to expect that linear or piecewise linear transformations should be valid transformations for the other groups too. As the size of our dataset was too small to enable reliable estimation of piecewise linear transformations, we restricted ourselves to linear ones. However, piecewise linear transformations would be worthy of investigation in studies in which more datapoints are available.

The form of nonlinearity in (3) enabled us to describe lung function decline in adulthood quite accurately whilst keeping the model relatively simple. However, it would be worthy to also consider the more flexible case in which the form is estimated, particularly when considering other types of measurement in addition/replacement to FEV_1_ and FVC. Some work in this direction, specifically addressing complex lung function growth in young individuals, has been done in [18] and in [16], which proposed the model of [28]. We are currently investigating modelling lung function decline with Gaussian radial basis functions.

Treating *b* as deterministic rather than random enabled us to use simple numerical integration for inference, avoiding the need to develop more complex approximation schemes. It is reasonable to assume that a posterior on *b* would be highly peaked (as is the posterior of *u*_1_,*v*_1_, *p*(*u*_1_,*v*_1_|{*l*^*n*^|*c*^*n*^=1}), computed from the same individuals) and therefore that this choice had minor impact on the estimated uncertainties.

Finally, we would like to notice that, whilst the proposed model can also provide single individuals with biological age, such a usage of the model would require a careful analysis on how to set the measurement noise covariance *Σ*_*l*_, as the maximum likelihood approach used in this paper could be a suboptimal.

## Conclusions

We have introduced a probabilistic model based on the concept of biological age to analyse the effects of smoking, asthma and COPD on female lung function. Our approach enabled us to make statements over large age ranges and about co-occurrence of factors with little data.

We have found that co-occurrence of smoking with asthma or COPD or combined asthma and COPD has more severe effect on ageing than smoking, asthma, COPD or combined asthma and COPD alone. This is in agreement with the findings in [29], that suggest that the rate of decline of lung function is faster in smokers with emphysema than in ex-smokers with emphysema. This is also in line with the results in [4,20,30], which show that smoking has a strong additional ageing effect on individuals with asthma. To the best of our knowledge, results on co-occurrence of smoking with combined asthma and COPD have not been previously reported.

We have also found that co-occurrence of asthma and COPD has a more detrimental effect on the lungs than asthma or COPD alone. This is in line with recent studies that indicate a reduced quality of life in individuals with both asthma and COPD with respect to individuals that have only either disease [31-33].

By analysing differences among ages, we could conclude that, with the exception of asthma and COPD alone, the effects of the combinations of factors increase with age and therefore are more severe at older ages. This is in agreement with other studies, for example [4], in which it is shown that the effects of smoking and combined smoking with asthma increase with age, whilst the effect of asthma is constant.

At age 50 for which the standard deviations are lower, our model estimated that the average number of years ±1× the standard deviations added to chronological age by the factors are approximately as follows. Asthma: 6.6 ±1.4; COPD: 5.7 ±4.0; asthma-COPD: 8.8 ±3.5; smoking: 6.6 ±0.7; smoking-asthma: 16.8 ±3.5; smoking-COPD: 17.2 ±2.0; smoking-asthma-COPD: 29.5 ±3.0.

The software implementing the model can be downloaded at the first author’s webpage.

## Appendix

Below we describe how to estimate the model parameters *b*, $\sigma_{ã}^{2}$ and *Σ*_*l*_ and the posterior distributions *p*(*u*_*j*_,*v*_*j*_|{*l*^*n*^|*c*^*n*^=*j*}) and *p*(*u*_*j*_*a*+*v*_*j*_|{*l*^*n*^|*c*^*n*^=*j*}). In order to avoid underflow/overflow problems, computations were performed in log-scale.

### Parameter learning

As explained above, the parameter set *b* was estimated from the healthy group (Group H) only to make sure that it describes lung function decline in the absence of smoking, asthma and COPD. We learned the two subsets of *b* corresponding to FEV_1_ and FVC separately using ordinary least squares. We then fixed *b* and estimated parameters $\sigma_{ã}^{2}$ and *Σ*_*l*_ using an Expectation Maximization (EM) approach [34]. More specifically, the EM approach consisted of iterating the following two steps until convergence: 

•E-Step: Perform inference on *p*(*ã*^1^,…,*ã*^*N*^,*u*_1_…,*u*_8_,*v*_1_,…,*v*_8_|*l*^1^,…,*l*^*N*^) to compute the quantities required to perform the M-Step.

•M-Step: Find the values of $\sigma_{ã}^{2}$ and *Σ*_*l*_ that maximize the expectation of the complete data log-likelihood

$$\begin{array}{ll} {arg max}_{\sigma_{ã}^{2},\Sigma_{l}}{\langle \log p(l^{1},\ldots ,l^{N},{ã}^{1},\ldots ,{ã}^{N},u_{1}\ldots ,u_{8},v_{1},\ldots ,v_{8})\rangle }_{p({ã}^{1},\ldots ,{ã}^{N},u_{1}\ldots ,u_{8},v_{1},\ldots ,v_{8}|l^{1},\ldots ,l^{N})}\;, & \; \end{array}$$

•where 〈·〉_*p*(·)_ denotes averaging with respect to *p*(·) and *p*(*ã*^1^,…,*ã*^*N*^,*u*_1_…,*u*_8_,*v*_1_,…,*v*_8_|*l*^1^,…,*l*^*N*^) is computed using the values of $\sigma_{ã}^{2}$ and *Σ*_*l*_ estimated in the previous iteration.

The part of the expectation of the complete data log-likelihood that depends on $\sigma_{ã}^{2}$ and *Σ*_*l*_ is given by

(4) $$\begin{array}{ll} \underset{j}{\sum }\underset{\left\{n|c^{n}=j\right\}}{\sum }\left[{\langle \log p(l^{n}|{ã}^{n})\rangle }_{p({ã}^{n}|\{l^{n^{\prime }}|c^{n^{\prime }}=j\})}+{\langle \log p({ã}^{n}|u_{j},v_{j})\rangle }_{p({ã}^{n},u_{j},v_{j}|\{l^{n^{\prime }}|c^{n^{\prime }}=j\})}\right]\;. & \; \end{array}$$

We excluded the parameter set *b* from the EM approach as we found that otherwise the nonlinearity in FEV_1_ and FVC decline with age of healthy individuals would be transferred to the biological age (through high noise variance $\sigma_{ã}^{2}$) so that *b* would not represent normal lung function decline.

#### M-Step: Updates for

$$\sigma_{ã}^{2}$$

Setting to zero the derivative of (4) with respect to $\sigma_{ã}^{2}$

$$\begin{array}{ll} \underset{j}{\sum }\underset{\left\{n|c^{n}=j\right\}}{\sum }{\langle \frac{\partial \log p({ã}^{n}|u_{j},v_{j})}{\partial \sigma_{ã}^{2}}\rangle }_{p({ã}^{n},u_{j},v_{j}|\{l^{n^{\prime }}|c^{n^{\prime }}=j\})}\propto -N+\underset{j}{\sum }\underset{\left\{n|c^{n}=j\right\}}{\sum }\frac{{\langle {\left({ã}^{n}-u_{j}a^{n}-v_{j}\right)}^{2}\rangle }_{p({ã}^{n},u_{j},v_{j}|\{l^{n^{\prime }}|c^{n^{\prime }}=j\})}}{\sigma_{ã}^{2}}\;, & \; \end{array}$$

we obtain the optimal $\sigma_{ã}^{2}$

$$\begin{array}{lll} & \sigma_{ã}^{2}=\frac{1}{N}\underset{j}{\sum }\underset{\left\{n|c^{n}=j\right\}}{\sum }\left(\langle {\left({ã}^{n}\right)}^{2}\rangle +\langle u_{j}^{2}\rangle {\left(a^{n}\right)}^{2}+\langle v_{j}^{2}\rangle -2\langle {ã}^{n}u_{j}\rangle a^{n}-2\langle {ã}^{n}v_{j}\rangle +2\langle u_{j}v_{j}\rangle a^{n}\right)\;,\; & \; \end{array}$$

where the required moments are estimated as explained below.

#### *M-Step: Updates forΣ*_*l*_

Setting to zero the derivative of (4) with respect to $\Sigma_{l}^{-1}$

$$\begin{array}{ll} \underset{j}{\sum }\underset{\left\{n|c^{n}=j\right\}}{\sum }{\langle \frac{\partial \log p(l^{n}|{ã}^{n})}{\partial \Sigma_{l}^{-1}}\rangle }_{p({ã}^{n}|\{l^{n^{\prime }}|c^{n^{\prime }}=j\})}\propto N\Sigma_{l}-\underset{j}{\sum }\underset{\left\{n|c^{n}=j\right\}}{\sum }{\langle \left({\tilde{l}}^{n}-b_{2}{ã}^{n}-b_{3}{\left({ã}^{n}\right)}^{2}\right){\left({\tilde{l}}^{n}-b_{2}{ã}^{n}-b_{3}{\left({ã}^{n}\right)}^{2}\right)}^{\text{T}}\rangle }_{p({ã}^{n}|\{l^{n^{\prime }}|c^{n^{\prime }}=j\})}\;, & \; \end{array}$$

where ${\tilde{l}}^{n}=l^{n}-b_{1}-b_{4}\log h^{n}$, we obtain the optimal *Σ*_*l*_

$$\begin{array}{lll} \Sigma_{l}=\frac{1}{N}\underset{j}{\sum }\underset{\left\{n|c^{n}=j\right\}}{\sum }\left[\right) & {\tilde{l}}^{n}{\left({\tilde{l}}^{n}\right)}^{\text{T}}-\langle {ã}^{n}\rangle ({\tilde{l}}^{n}b_{2}^{\text{T}}+b_{2}{\left({\tilde{l}}^{n}\right)}^{\text{T}})-\langle {\left({ã}^{n}\right)}^{2}\rangle \left(\right){\tilde{l}}^{n}b_{3}^{\text{T}}+b_{3}{\left({\tilde{l}}^{n}\right)}^{\text{T}}-b_{2}b_{2}^{\text{T}}))\; & \;\\ & +\langle {\left({ã}^{n}\right)}^{3}\rangle (b_{2}b_{3}^{\text{T}}+b_{3}b_{2}^{\text{T}})+\langle {\left({ã}^{n}\right)}^{4}\rangle b_{3}b_{3}^{\text{T}}])\;.\; & \; \end{array}$$

#### *E-Step: Inference onp*(*ã*^1^,…,*ã*^*N*^,*u*_1_…,*u*_8_,*v*_1_,…,*v*_8_|*l*^1^,…,*l*^*N*^)

The marginal likelihood can be estimated as

$$\begin{array}{lll} p(l^{1:N}) & =\underset{j}{\prod }p(\{l^{n}|c^{n}=j\})=\underset{j}{\prod }\underset{u_{j},v_{j}}{\int }\left[\overset{p_{j}}{\overset{︷}{\underset{\left\{n|c^{n}=j\right\}}{\prod }p(l^{n}|u_{j},v_{j})}}\right]p(u_{j})p(v_{j})\; & \;\\ & =\underset{j}{\prod }\underset{u_{j},v_{j}}{\int }\left[\underset{\left\{n|c^{n}=j\right\}}{\prod }\underset{{ã}^{n}}{\int }p(l^{n},{ã}^{n}|u_{j},v_{j})\right]p(u_{j})p(v_{j})\; & \;\\ & =\underset{j}{\prod }\underset{u_{j},v_{j}}{\int }\left[\underset{\left\{n|c^{n}=j\right\}}{\prod }\underset{{ã}^{n}}{\int }p(l^{n}|{ã}^{n})p({ã}^{n}|u_{j},v_{j})\right]p(u_{j})p(v_{j})\; & \;\\ & =\underset{j}{\prod }\underset{u_{j},v_{j}}{\int }\left[\underset{\left\{n|c^{n}=j\right\}}{\prod }\underset{{ã}^{n}}{\int }N(b_{1}+b_{2}{ã}^{n}+b_{3}{\left({ã}^{n}\right)}^{2}+b_{4}\log h^{n},\Sigma_{l})N(u_{j}a^{n}+v_{j},\sigma_{ã}^{2})\right]p(u_{j})p(v_{j})\;,\; & \; \end{array}$$

where the required integrations are computed numerically.

Then the posterior distribution $p({ã}^{n},u_{j},v_{j}|\{l^{n^{\prime }}|c^{n^{\prime }}=j\})$ can be estimated as

(5) $$\begin{array}{lll} p({ã}^{n},u_{j},v_{j}|\{l^{n^{\prime }}|c^{n^{\prime }}=j\}) & =\frac{p({ã}^{n},u_{j},v_{j},\{l^{n^{\prime }}|c^{n^{\prime }}=j\})}{p(\{l^{n^{\prime }}|c^{n^{\prime }}=j\})}\; & \;\\ & =\frac{p({ã}^{n},u_{j},v_{j},l^{n})\underset{\left\{n^{\prime }|n^{\prime }\neq n,c^{n^{\prime }}=j\right\}}{\prod }p(l^{n^{\prime }}|u_{j},v_{j})}{p(\{l^{n^{\prime }}|c^{n^{\prime }}=j\})}\; & \;\\ & =\frac{p(l^{n}|{ã}^{n})p({ã}^{n}|u_{j},v_{j})p(u_{j})p(v_{j})p_{j}}{p(l^{n}|u_{j},v_{j})p(\{l^{n^{\prime }}|c^{n^{\prime }}=j\})}\;.\; \end{array}$$

From this distribution, the moments required for the parameter updates, namely 〈*ã*^*n*^〉, 〈(*ã*^*n*^)^2^〉, 〈(*ã*^*n*^)^3^〉, 〈(*ã*^*n*^)^4^〉, 〈*ã*^*n*^*u*_*j*_〉, 〈*ã*^*n*^*v*_*j*_〉, $\langle u_{j}^{2}\rangle$, $\langle v_{j}^{2}\rangle$ and 〈*u*_*j*_*v*_*j*_〉, are computed by numerical integration.

#### Approximation

The EM approach for learning $\sigma_{ã}^{2}$ and *Σ*_*l*_ described above is time consuming. A comparison of this approach with an approximation in which *u*_*j*_ and *v*_*j*_ are considered as deterministic did not show any difference in the learned values of $\sigma_{ã}^{2}$ and *Σ*_*l*_. We therefore used this approximation for the presented results.

In this alterative approach, the updates for $\sigma_{ã}^{2}$ and *Σ*_*l*_ in the M-Step are similar to the ones above in which the optimal values of *u*_*j*_ and *v*_*j*_ are used and $p({ã}^{n},u_{j},v_{j}|\{l^{n^{\prime }}|c^{n^{\prime }}=j\})$ becomes $p({ã}^{n}|l^{n})$, computed as $p(l^{n}|{ã}^{n})p({ã}^{n})/\underset{{ã}^{n}}{\int }p(l^{n}|{ã}^{n})p({ã}^{n})$. The optimal values of *u*_*j*_ and *v*_*j*_ are learned by setting to zero

$$\begin{array}{lcrr} & \underset{\left\{n|c^{n}=j\right\}}{\sum }{\langle \frac{\partial \log p({ã}^{n})}{\partial u_{j}}\rangle }_{p({ã}^{n}|l^{n})}\propto \underset{\left\{n|c^{n}=j\right\}}{\sum }\left({\langle {ã}^{n}\rangle }_{p({ã}^{n}|l^{n})}-u_{j}a^{n}-v_{j}\right)a^{n}\;, & & \\ & \underset{\left\{n|c^{n}=j\right\}}{\sum }{\langle \frac{\partial \log p({ã}^{n})}{\partial v_{j}}\rangle }_{p({ã}^{n}|l^{n})}\propto \underset{\left\{n|c^{n}=j\right\}}{\sum }\left({\langle {ã}^{n}\rangle }_{p({ã}^{n}|l^{n})}-u_{j}a^{n}-v_{j}\right)\;, & & \end{array}$$

that is, by solving the following linear system:

$$\begin{array}{lcrr} \;\;\;\left(\begin{array}{ll} \underset{\left\{n|c^{n}=j\right\}}{\sum }{\left(a^{n}\right)}^{2} & \underset{\left\{n|c^{n}=j\right\}}{\sum }a^{n}\\ \underset{\left\{n|c^{n}=j\right\}}{\sum }a^{n} & N_{j} \end{array}\right)\left(\begin{array}{l} u_{j}\\ v_{j} \end{array}\right)=\left(\begin{array}{l} \underset{\left\{n|c^{n}=j\right\}}{\sum }{\langle {ã}^{n}\rangle }_{p({ã}^{n}|l^{n})}a^{n}\\ \underset{\left\{n|c^{n}=j\right\}}{\sum }{\langle {ã}^{n}\rangle }_{p({ã}^{n}|l^{n})} \end{array}\right)\;, & & & \end{array}$$

where *N*_*j*_ indicates the number of measurements belonging to Group *j*.

### Computing the effects of smoking, asthma and COPD

The posteriors distributions *p*(*u*_*j*_,*v*_*j*_|{*l*^*n*^|*c*^*n*^=*j*}) can be computed from (5) by numerical integration over *ã*^*n*^. The posteriors distributions *p*(*u*_*j*_*a*+*v*_*j*_|{*l*^*n*^|*c*^*n*^=*j*}) can be computed from *p*(*u*_*j*_,*v*_*j*_|{*l*^*n*^|*c*^*n*^=*j*}) using the formula of linear transformation of random variables and numerical integration. However, as we found numerically that *p*(*u*_*j*_,*v*_*j*_|{*l*^*n*^|*c*^*n*^=*j*}) are Gaussian, *p*(*u*_*j*_*a*+*v*_*j*_|{*l*^*n*^|*c*^*n*^=*j*}) can be computed more simply using the formula of linear transformation of Gaussian random variables. A transformation of *p*(*u*_*j*_,*v*_*j*_|{*l*^*n*^|*c*^*n*^=*j*}) was performed to correct the small deviation of the mean of *p*(*u*_1_,*v*_1_|{*l*^*n*^|*c*^*n*^=1}) from (1,0).

## Abbreviations

COPD: Chronic obstructive pulmonary disease; FEV1: Forced expiratory volume in one second; FVC: Forced vital capacity.

## Competing interests

The authors declare that they have no competing interests.

## Authors’ contributions

SC conceived and implemented the model, performed the experiments, the data filtering, and wrote the manuscript. JW contributed to the discussion and interpretation of the model and of the experiments and revised and gave suggestions about the structure of the manuscript. AV contributed to the discussion and interpretation of the model and of the experiments and revised the manuscript. HT performed data cleaning and smoking, asthma and COPD status assignment. TS contributed to data experimental design and collection. All authors read and approved the final manuscript.
